# Data on the effect of *in vivo* knockdown using artificial ErbB3 miRNA on Remak bundle structure

**DOI:** 10.1016/j.dib.2017.04.014

**Published:** 2017-04-15

**Authors:** Yuki Miyamoto, Tomohiro Torii, Kazuko Kawahara, Masashi Inoue, Takako Morimoto, Masahiro Yamamoto, Junji Yamauchi

**Affiliations:** a Laboratory of Molecular Neuroscience and Neurology, School of Life Sciences, Tokyo University of Pharmacy and Life Sciences, Hachioji, Tokyo 192-0355, Japan; b Department of Pharmacology, National Research Institute for Child Health and Development, Setagaya, Tokyo 157-8535, Japan; c Department of Neuroscience, Baylor College of Medicine, Houston, TX 77030, USA; d Tsumura Research Laboratories, Tsumura & Co., Inashiki, Ibaraki 200-1192, Japan

**Keywords:** ErbB3, Knockdown, miRNA, Remark bundle, Sciatic nerve

## Abstract

Mature Schwann cells, the peripheral nervous system (PNS) glial cells, have two major roles for neuronal axons (Bunge, 1993) [1]. For large diameter axons, Schwann cells form myelin sheaths with multiple layers. For small diameter axons, they form Remak bundle composed only of single layer of the Schwann cell plasma membrane. In the PNS, ErbB3 forms a dimer with ErbB2 on the Schwann cell plasma membrane. ErbB3 plays a key role in myelination by myelinating Schwann cells, that is to say, its role in myelin thickness. Herein we provide the data regarding the effect of *in vivo* knockdown of ErbB3 on the thickness between an axon and a neighboring axon in Remak bundle, which is formed by non-myelinating Schwann cells. Since ErbB3 knockout mice are embryonically lethal, Schwann cell lineage-specific transgenic mice transcribing ErbB3 shRNA with an artificial miRNA backbone were generated and used in these experiments (Torii et al., 2014) [2].

**Specifications Table**

**Table:** 

Subject area	Biology
More specific subject area	Neurobiology, molecular and cellular neuroscience, gene-modifying technology
Type of data	Figure
How data was acquired	Electron microscopy
Data format	Raw data, analyzed data
Experimental factors	ErbB3 knockdown mice were used for experiments.
Experimental features	Electron microscopic analysis
Data source location	Laboratory of Molecular Neuroscience and Neurology, School of Life Sciences, Tokyo University of Pharmacy and Life Sciences, Tokyo, Japan
Data accessibility	Data is available with this article

**Value of the Data**

• This data set is of value to the scientific community to need the information for the biological effect of a receptor tyrosine kinase.
• The data provide the valuable information for the role of a receptor tyrosine kinase in the nervous system.
• The data allow us to promote our understanding of how a receptor tyrosine kinase contributes to Schwann cell development, especially to the thickness between an axon and a neighboring axon in Remak bundle.
• The data provide the valuable information for analyzing the role of target molecules using transgenic mice.

## 1 Data

The data shared in this article provide electron microscopic analyses of sciatic nerve׳s Schwann cells in Schwann cell lineage-specific ErbB3 shRNA transgenic mice (ErbB3 knockdown mice). The data shared also provide electron microscopic analyses of neuronal fibers in transgenic mice.

## 2 Experimental design, materials and methods

### 2.1 Data from ErbB3 shRNA transgenic mice

**Fig. 1:**
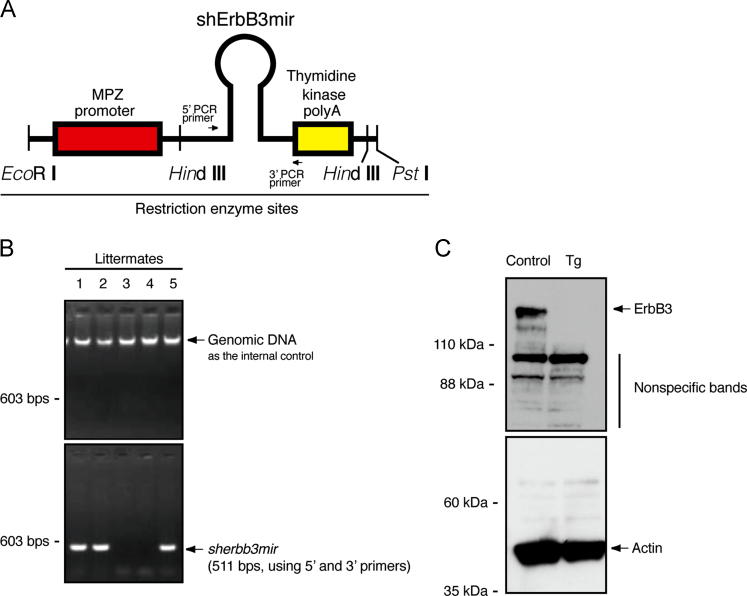
Construction of ErbB3 shRNA transgene. (A) Construction of ErbB3 shRNA transgene. Genomic primer positions and restriction enzyme sites are shown. Mouse MPZ promoter, shErbB3mir, and BLOCK-iT vector-derived polyA signal are also shown. (B) Control genemic DNA and genomic PCR data are shown. In this PCR test, Nos. 1, 2, and 5 mice are transgenic (Tg) ones. (C) Sciatic nerve lysates were immunoblotted with antibodies against ErbB3 and control actin.

**Fig. 2:**
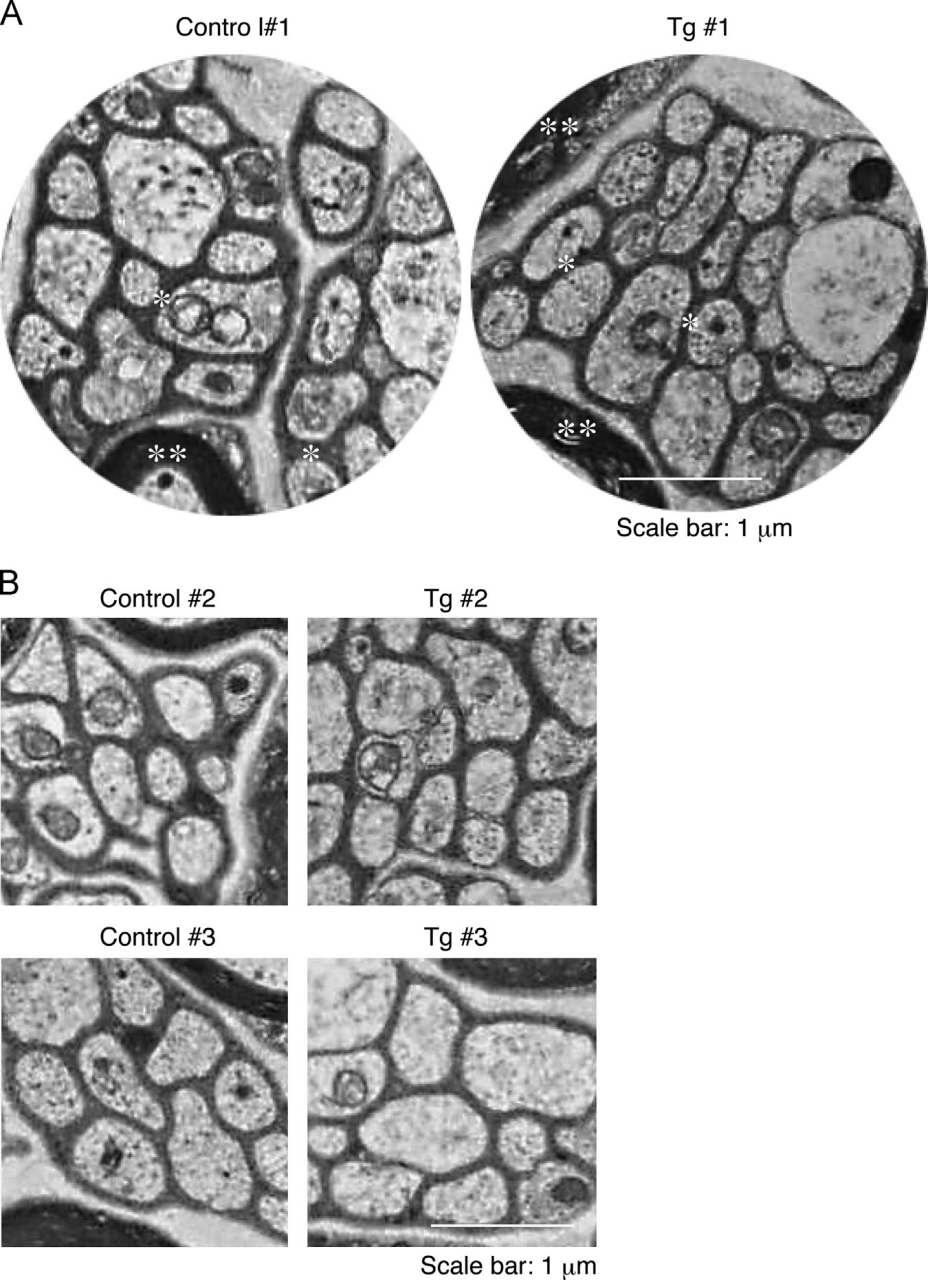
Electron microscopic images (low magnification) of ErbB3 shRNA transgenic mice and littermate controls. (A) Electron microscopic images (2500-fold) of cross sections in ErbB3 shRNA transgenic mice (Tg#1, right image) and littermate controls (control#1, left image) are shown. Single and double asterisks indicate Schwann cell plasma membrane-derived Remak bundle and compact myelin, respectively. Scale bar indicates 1 μm. (B) Electron microscopic images in two other mice (#2 and #3) are also shown. Scale bar indicates 1 μm.

**Fig. 3:**
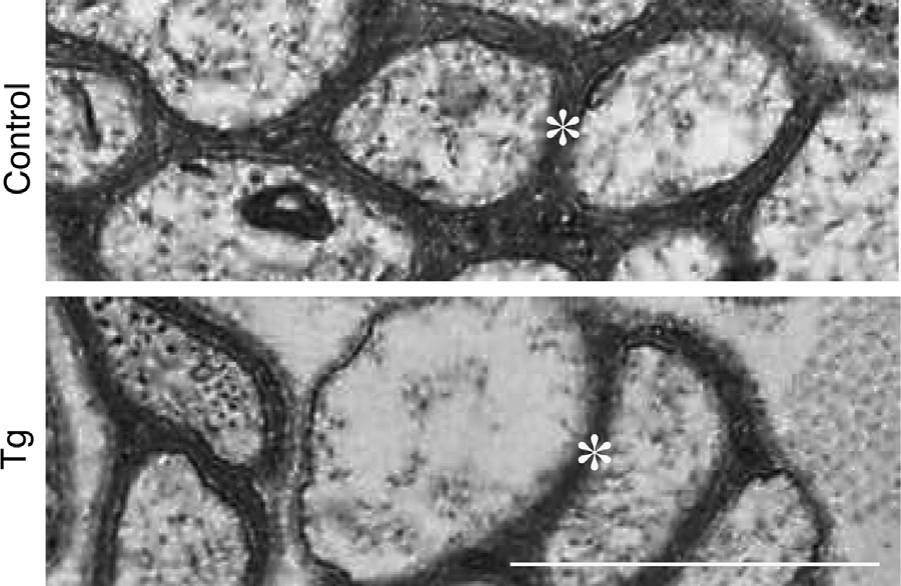
Electron microscopic images (high magnification) of ErbB3 shRNA transgenic mice and littermate controls. Representative electron microscopic images (5000-fold) of cross sections in ErbB3 shRNA transgenic mice (Tg, lower image) and littermate controls (control, upper image) are shown. Single asterisks indicate Schwann cell plasma membrane-derived Remak bundle. Scale bar indicates 1 μm.

**Fig. 4:**
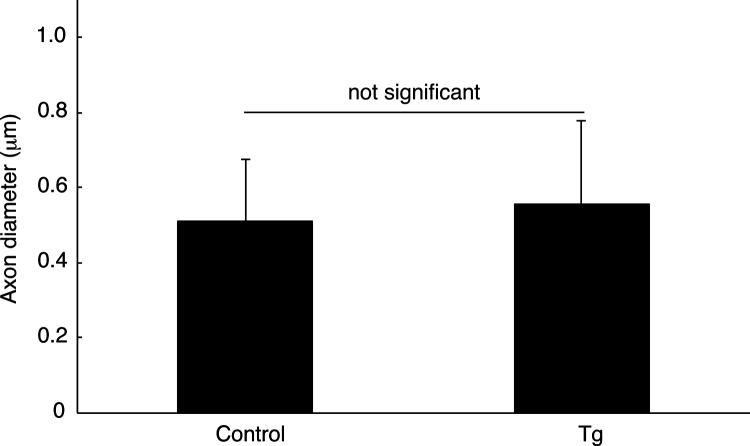
Comparison of the length of axon diameters in Remak bundles in ErbB3 shRNA transgenic mice and the controls. The length of axon diameters in Remak bundles of ErbB3 shRNA transgenic mice (Tg) and the controls (control) was measured (not significant, p=0.657, not significant; n=9 of three independent mice; unpaired Student׳s *t*-test).

**Fig. 5:**
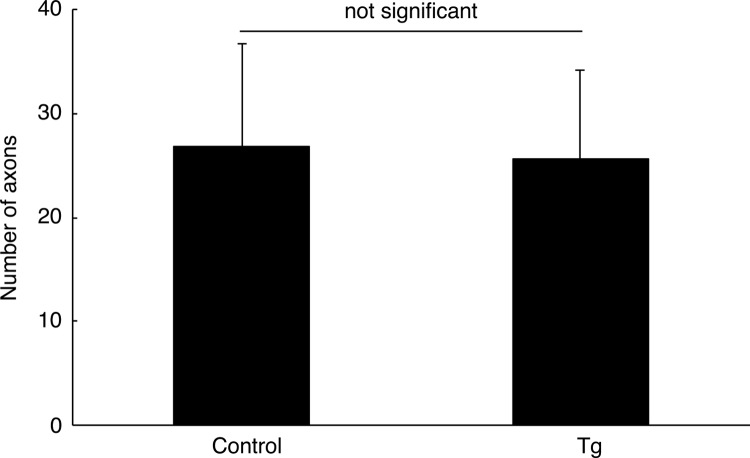
Comparison of the number of axons in Remak bundles in ErbB3 shRNA transgenic mice and the controls. The number of axons in Remak bundles of ErbB3 shRNA transgenic mice (Tg) and the controls (control) was measured (not significant, *p*=0.846, not significant; *n*=6 of three independent mice; unpaired Student׳s *t*-test).

**Fig. 6:**
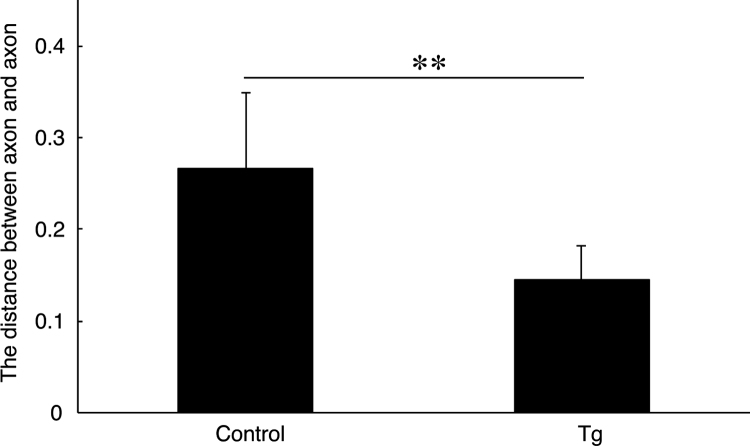
Comparison of the distance between an axon and a neighboring axon in Remak bundles in ErbB3 shRNA transgenic mice and the controls. The distance between an axon and a neighboring axon in Remak bundles of ErbB3 shRNA transgenic mice (Tg) and the controls (control) was measured (**, *p*=0.00138; *n*=9 of three independent mice; unpaired Student׳s *t*-test).

While myelin is composed of multiple layer plasma membranes of myelinating Schwann cells, Remak bundle is axonal one with single layer of the plasma membranes of non-myelinating Schwann cells [1]. In Schwann cell lineage-specific ErbB3 shRNA transgenic mice (Fig. 1; Ref. [2]) and littermate controls, axon diameter (Fig. 2, Fig. 3, Fig. 4) and axon number (Fig. 2, Fig. 3; Fig. 5) in Remak bundle were comparable. On the other hand, in ErbB3 shRNA transgenic mice, the distance between an axon and a neighboring axon was significantly short (Fig. 2, Fig. 3; Fig. 6), showing the role of Schwann cell ErbB3 in Remak bundle formation.

### 2.2 ErbB3 shRNA transgenic mice

The mouse Schwann cell-specific MPZ promoter (GenBank Acc. No. M62857) was amplified using C57BL/6J mouse genomic DNAs. Mouse ErbB3 shRNAs, designed using an RNAi Design program (http://rnaidesigner.lifetechnologies.com/rnaiexpress/), were inserted into the BLOCK-iT PolII miR RNAi expression vector (Cat. No. K4936-00; Life Technologies), followed by amplification with the 704–2010 bases. The ErbB3 nucleotide target sequences used were 5′-TACCCATGACCACCTCACACT-3′ (ErbB3׳s 164–184 bases) and 5′-ATATCTGGCAGTCTTCTGGTC-3′ (ErbB3׳s 593–613 bases) [2]. The nucleotides encoding the promoter, shRNA-inserted artificial miRNA (tentatively called shErbB3mir), and BLOCK-iT vector-derived polyA signal units were successively inserted into the pCMV5 backbone as the subcloning vector. A DNA fragment (~2.7-kb) containing all nucleotide units was digested from the vector backbone with *Eco*RI and *Pst*I, purified, and injected into fertilized C57BL/6J oocytes [2], [3], [4]. Transgenic mice were identified by tail DNA׳s genomic PCR with specific primers (5′ primer, 5′-GCTAAGCACTTCGTGGCCGTCGATCG-3′; and 3′ primer, 5′-GCGAGCCCTGGGCCTTCACC-3′ for the artificial miRNA sequence), showing 511 bps. PCR was performed in 30 cycles, each consisting of denaturation at 94 °C for 1 min, annealing at 65 °C for 1 min, and extension at 72 °C for 1 min. One transgenic line׳s mice were mated to wild type C57BL/6J mice and were fertile under standard breeding conditions. Male mice were used for experiments if it was possible to distinguish their sex.

### 2.3 Electron microscopy

Sciatic nerves (2-month-old) were fixed with 2% paraformaldehyde and 2% glutaraldehyde in 0.1% cacodylate buffer. The tissues were postfixed with buffered 2% osmium tetroxide, dehydrated with an ethanol gradient, treated with acetone, and embedded in epoxy resin. Ultrathin cross sections, using Cryostar NX70 (Thermo Fisher Scientific), were stained with uranyl acetate and lead citrate. They were observed and photographed with Hitachi electron microscopes [5]. In order to unify the morphologies of Remak bundles in cross sections, we sliced middle portions of sciatic nerves in three mice and calculated the statistical data.

### 2.4 Immunoblotting

The lysates from 2-month-old sciatic nerve tissues were denatured and then separated on sodium dodecyl sulfate-polyacrylamide gels. The electrophoretically separated proteins were transferred to PVDF membranes, blocked with Blocking One reagent (Nacalai Tesque), and immunoblotted first with primary antibodies and then with peroxidase-conjugated secondary antibodies. The bound antibodies were detected using Nacalai Tesque׳s chemiluminescence reagent. Anti-ErbB3 and anti-actin (control actin) antibodies were obtained from Cell Signaling Technology and MBL, respectively. At least three experiments were carried out under each condition, and a representative bot is shown in the figure.

### 2.5 Ethics statement

Genetically modified/unmodified mice were maintained in accordance with a protocol approved by the Japanese National Research Institute for Child Health and Development Animal Care Committee. They were also maintained in accordance with a protocol approved by the Tokyo University and Pharmacy and Life Sciences Animal Care Committee.
